# Food Myths or Food Facts? Study about Perceptions and Knowledge in a Portuguese Sample

**DOI:** 10.3390/foods10112746

**Published:** 2021-11-09

**Authors:** Sofia G. Florença, Manuela Ferreira, Inês Lacerda, Aline Maia

**Affiliations:** 1 Faculty of Food and Nutrition Sciences, University of Porto, 4150-180 Porto, Portugal; 2 UICISA:E, Polytechnic Institute of Viseu, 3504-510 Viseu, Portugal; manuelaferreira@sc.ipv.pt; 3 ACES Dão-Lafões, Grouping of Health Centres of Dão-Lafões, 3514-511 Viseu, Portugal; IMLacerda@arscentro.min-saude.pt (I.L.); ADMaia@arscentro.min-saude.pt (A.M.)

**Keywords:** food myths, sources of information, nutritional knowledge, health

## Abstract

Food myths are nutritional concepts poorly justified or even contradict existing scientific evidence that individuals take as the truth. Knowledge in nutrition is an important tool in tackling misinformation and in the promotion of adequate food choices. This study aimed to investigate the beliefs and perceptions of a sample of the Portuguese population regarding a series of food myths and facts, evaluating, consequently, the level of knowledge and the main sources of information. The research was conducted on a sample of 503 participants, using a questionnaire disclosed online, by email, and social networks, between May and June of 2021. Thirty statements, some true and others false, were analyzed to assess people’s perceptions. Based on the respondents’ answers, a score was calculated for each statement, allowing to differentiate the correct (positive score) from incorrect (negative score) perceptions. The results showed that most statements obtained positive scores, corresponding to correct perceptions. Moreover, the level of knowledge was measured, being very high for 21.7% of the participants and high for 42.1%. The main sources where the participants acquire nutritional information are scientific journals (43.3%), website of the Portuguese General Health Office (DGS) (31.4%), and technical books (31.0%), which is concordant with the level of trust in these sources. Hence, it was concluded that, despite the levels of nutritional knowledge, there are still several food myths that need to be debunked, through the proper channels, in order to promote healthy, balanced, and adequate eating behaviors.

## 1. Introduction

Food is one of the basic necessities of humans, being fundamental in the promotion of health and well-being, as well as the prevention of diseases [1]. However, even though food consumption has the main goal of satisfying a physiological need, hunger, other factors also determine what is the motivation, quantity, frequency, and food choice. Some of these factors are related to appetite, cost, accessibility, emotions, culture, and social interactions [2,3]. Food choices are, therefore, an overlap of several domains, from exact sciences, for example nutrition, to social policies and the individual behavior [1]. A work by Milošević et al. [4] carried out in six Balkan countries, based on the Food Choice Questionnaire, allowed identifying eight factors underlying food choice and five groups of consumers according to their motivational profiles. Hence, the authors found the participants distributed by groups depending on their food purchasing behavior and socioeconomic characteristics. Cunha et al. [5] published a review about the application of the Food Choice Questionnaire in different countries, and they concluded that different contexts influence people’s responses and, therefore, even using the same instruments, the results might be differentiated among countries. Guiné et al. [6] also investigated the eating motivations in 16 countries and found them related to different types of factors: health, emotions, price and availability, society and culture, environment and politics, and marketing and commercials.

Beliefs are one of the many factors that influence eating behaviors, arising from a set of concepts adopted by an individual as the truth. Food myths are beliefs; that is, nutritional concepts poorly justified or even contradicting existing scientific evidence that are, however, taken as the truth by the individual [2,7]. Many of the existing myths are born from the current misinformation, and it is this lack of trustworthy information and knowledge about nutrition that results, most of the time, in diseases such as the metabolic syndrome, hypertension, diabetes, atherosclerosis, and cardiovascular diseases [2]. However, can eating behavior really be influenced by nutritional knowledge? The main goal of numerous food education programs is to improve nutritional knowledge, contributing to the establishment and maintenance of healthy, balanced, and varied eating habits. A review by Worsley [7] highlighted the implications of the nutritional knowledge for public health, as follows: develop more proactive educational programs, attribute greater importance to the education of children and adults, consider people’s personal food motivations, and continue developing research that allows better understanding consumer trends, and design appropriate interventions. According to another systematic review by Spronk et al. [8], nutritional knowledge is positively correlated, even if weakly, to food consumption, and so, the promotion of nutritional literacy should be considered a relevant nutritional policy to the modification of eating behaviors.

The lack of knowledge about certain food items can be related to their own perception and misperception of the information available. Hence, food items could be perceived as good when in fact, they are not, mainly due to cognitive errors. Marsola et al. [9] evaluated the perceptions of Brazilian consumers about the risks and benefits of different foods and how they perceived their related health effects. They concluded that income, education, and the absence of children are factors that contribute to increased perception of the health benefits as compared to the risks. Additionally, nutritional knowledge, is, many times, obtained through unreliable information sources [10]. A Canadian study has shown that, between 2004 and 2008, the primary sources of information about nutrition and food were food labels, followed by magazines/journals/books, colleagues/friends/family, internet, and health care workers. When evaluating the level of credibility provided by the sources, nutritionists and health professionals were the ones who obtained a better level of credibility [10]. Nutrition knowledge is an important tool in tackling misinformation and in the promotion of adequate eating behaviors. However, the information sources must be reliable and trustworthy, hence the primary role of health professionals in educating and monitoring the food habits of the population in a country, thus allowing the control of various chronic pathologies influenced by lifestyle [10]. According to the Global Burden of Disease report [11], from 2017, inadequate food habits are responsible for 11 million deaths every year, from which the main cause of death are diseases intrinsically linked to maladjusted eating behaviors, such as cardiovascular diseases, cancer, and type 2 diabetes. These data corroborates the existence of a new global pandemic of chronic diseases that are directly connected with food [11,12]. Hence, nutrition has assumed a prominent role in the prevention, control, treatment, and even regression of several pathologies that are influenced by eating habits and behaviors, being important as a part of a healthy and balanced lifestyle [12,13].

In the scientific literature studies, it is possible to find the importance of food or nutritional literacy for improved health [14,15,16], how this literacy can be achieved [17,18,19], and what factors contribute to increase food literacy in the general population or among specific groups [20,21,22]. However, the scientific literature is lacking research studies focused on the myths related to human food consumption. Hence, there is a need to deepen the research into the wrong perceptions people sometimes have about some aspects related with food and eating, as well as the nutrients and their roles for human health. In this context, it is proposed to contribute to bringing some highlight into the matter of what are food myths and what are facts scientifically supported. The aim of this research was to investigate the beliefs and perceptions of a sample of the Portuguese population regarding a series of food myths and facts, evaluating, consequently, the level of knowledge. The main sources of information concerning nutritional knowledge were also analyzed, as well as their level of trust.

## 2. Materials and Methods

### 2.1. Instrument and Data Collection

The instrument used in this study was a questionnaire developed for this research. After preparing the questionnaire, and before its application, it was approved by the Ethical Commission at the Faculty of Food and Nutrition Sciences of University of Porto (reference N° 28/2021/CEFCNAUP/2021).

Since there were no previous studies related to this particular type of work to identify food myths in the literature, the questionnaire was based on a search previously made on the internet and blogs about nutrition to identify the main problematic topics. Additionally, the input from patients at appointments with nutritionists was also considered to design the questions. The questionnaire consisted of four parts. The first section collected data on sociodemographic data: age, sex, highest level of education completed, living environment, marital status, and area of professional activity. In the second section, some questions were included to obtain information regarding anthropometric data and lifestyle choices: height and weight (auto reported data), whether the participant is responsible for buying the food he eats, practicing a balanced diet, or the practice of specific food regimes. In the third section, thirty sentences were included, some of them true and others false (Table 1), to evaluate the level of concordance concerning some food myths and facts, assessing the sample’s perceptions on the subjects on a 5 point Likert scale: 1—strongly disagree, 2—disagree, 3—neutral, 4—agree, 5—strongly agree). Finally, the fourth section of the questionnaire consisted in the evaluation of the main sources of information and their level of trust: YouTube channels of influencers, blogs from nutritionists, website of the Portuguese General Health Office (DGS), website of the World Health Organization (WHO), shops of natural products, scientific journals, webpages, social networks, television, technical books, pharmacies, health centers, or appointments with medical doctors or nutritionists. The frequency of use of the different sources of information about nutritional facts was assessed in the scale: never, few times, many times. The scale for assessing the level of trust was: 1—no trust at all, 2—some trust, 3—much trust, 4—full trust.

The data were collected between May and June of 2021, and applied to a sample, chosen according to the facility of recruitment and willingness to participate. This survey was applied online through the Google Forms software and disclosed to people by different tools, such as email and social networks. Participation in the study was voluntary and only allowed for adults aged 18 years or older, but no upper age limit was established. All respondents gave their informed consent to participate and the confidentiality of the answers provided was guaranteed. The inclusion criteria to partake in the study, apart from age, was the access to internet, email, social networks, and a computer, as well as the skills necessary to be able to access the questionnaire. It guaranteed confidentiality of the responses by all participants, and these consented freely in their participation. Furthermore, they could abandon the questionnaire at any time without submitting the form. The design and application of the questionnaire has respected all ethical issues.

Considering the strategy used to collect the data, which occurred under COVID-19 limitations, the questionnaire was applied to a convenience sample, recruited according to facility of contact and disposition to take part in the research. Although convenience samples have some limitations, such as not allowing direct generalization of the conclusions from the sample to the whole population, they can also have some benefits, namely easiness of recruitment. Therefore they have been reported as a good tool to carry out research with exploratory nature [2,3]. Despite being a convenience sample, adequacy of the sample size was calculated, to serve as an indicator during data collection. For this, some assumptions were considered: confidence interval = 90%; Z score = 1.645; power of the test = 95% (minimum acceptable probability of preventing type II error = 0.05) [4,5]; Portuguese population in 2019 (the latest year available when the data collection started) = 10,283,822 people [6]: assumed that ~7.5 million were adults and the target population was 25% = 1875 thousand. Considering these conditions, calculation of the minimum sample size resulted in 203 adults [7,8]. The number of valid questionnaires obtained in this survey was 503, over the minimum number previously calculated.

### 2.2. Data Analysis

The statistical analysis was conducted using Excel 2016 and SPSS software V25 (IBM, Inc., Armonk, NY, USA). For treatment of data, basic statistical tools were used, such as frequencies, percentiles, mean values, and standard deviation.

For the data treatment, variable age was categorized into the following groups: young adults (18–30 years), middle-aged adults (31–50 years), senior adults (51–65 years), and elderly (aged 66 years or more). The body mass index (BMI) was calculated from the collected data as weight divided by squared height. The BMI was then classified according to the following categories of the WHO: underweight (<18.5 kg/m^2^), normal weight (18.5–24.9 kg/m^2^), overweight (25–29.9 kg/m^2^), and obesity (≥30 kg/m^2^). For those with BMI over 30 kg/m^2^, there are still differentiated classes of obesity; however, for the objectives of this study, these four classes were considered satisfactory enough [9,10,11]. The normality of the distribution was evaluated for some variables (age and BMI), testing the null hypothesis (H0: The data follow a normal distribution).

Spearman correlations (r_s_) were used to measure the association between some variables. The values of r_s_ varied between −1 and 1, with negative values corresponding to inverse associations between the variables. The reference absolute values considered were: r_s_ = 0—no correlation, r_s_ ∊ ]0.0, 0.2[—very weak correlation, r_s_ ∊ [0.2, 0.4[—weak correlation, r_s_ ∊ [0.4, 0.6[—moderate correlation, r_s_ ∊ [0.6, 0.8[—strong correlation, r_s_ ∊ [0.8, 1.0[—very strong correlation, r_s_ = 1—perfect correlation [12,13,14].

The agreement with the statements included in the questionnaire was used to calculate the scores for each item. However, before this, all of the incorrect statements (myths) were reversed to obtain the 30 items in the same scale of correct information. The variables were recoded from the 5-point Likert scale into the following scale: −1 = fully incorrect perception, −0.5 = partially incorrect perception, 0 = no opinion, 0.5 = partially correct perception, and 1 = fully correct perception. Then the scores for each of the 30 items were calculated as the sum of the values for each of the 503 participants. Hence, for each item, the sum score for perceptions varied between −503 to 503.

Additionally, the level of knowledge was calculated for each participant, as the sum of scores obtained for all items, after the previous operations of reversing the incorrect items and recoding into the scale from −1 to 1. In this way, for each participant, the sum varied between −30 and 30, and the values were then recoded into a categorical variable as class of knowledge: very low [−30;−15], low ]−15;0], medium ]0;10], high ]10;20], and very high knowledge ]20;30]. This variable was submitted to tree classification analysis for evaluation of the relative importance of the possible influential variables: sociodemographic variables (sex, age group, education, living environment, and marital status), other variables (job-nutrition, job-food, job-sport, job-health, BMI class, practice of balanced diet). A classification and regression tree (CRT) algorithm, with cross-validation and with minimum change in improvement of 0.0001, was used for treatment of the data, considering a limit of 5 levels and with a minimum number of cases in parent and child nodes equal to 30 and 15, respectively [15].

The variables frequency of use, of the different sources of information on nutritional facts and level of trust in those sources, were correlated by Spearman correlations.

The level of significance considered in the analyses was 5% (*p* < 0.05).

## 3. Results

### 3.1. Sample Characterization

In this survey, half of the participants were less than 44 years, with only 5% of the respondents under 21 years old, and 5% over 65 years old. It was further observed that 25% of participants were under 29 years old and 75% were over 52 years old. It was observed that age did not follow a normal distribution (*p* < 0.0005).

Regarding the sociodemographic characteristics (Table 2), the vast majority were middle-aged adults (31–50 years) (42.9%), women (77.8%), with university education (46.1%), living in urban environment (68.4%), and living in union or co-habiting (57.1%).

### 3.2. Lifestyle and Anthropometric Data

In this sample, most participants worked in professional areas related to nutrition (28.2%), health (33.4%), and food (34.8%) (Table 3). Of the people who answered the survey, 35.4% practiced caloric restriction and 8.5% flexitarianism (Table 3).

The acquisition of food was also questioned in this survey, with 88.3% of the participants being responsible for the purchase of the foods they consume.

The frequency of how often the respondents believe to be practicing a balanced and healthy diet was investigated based on the scale: always, frequently, sometimes, rarely, and never. The results showed that the majority, 52.5%, answered frequently, followed by 37% who answered sometimes, with only 5.6% acknowledging always, 4.4% rarely, and 0.6% never.

Some data were collected regarding the height and weight of those who partook in the study, in order to calculate BMI. It was found that BMI did not follow a normal distribution (*p* < 0.0005). There were 5% of the participants with a BMI under 19.1 kg/m^2^ and 5% with a BMI over 31.3 kg/m^2^ (Table 3). For this sample, 57.5% had normal weight (18.5–24.9 kg/m^2^) and 32.6% were overweight (25–29.9 kg/m^2^) (Table 3). A significant (*p* < 0.01) negative correlation was found between BMI and the self-reported practice of a balanced and healthy diet; however, the value was low (r_s_ = −0.271), meaning that the correlation was weak.

### 3.3. Perceptions Regarding Food Myths and Facts

Table 4 shows the items included in the survey related to different food myths and facts, and the corresponding sum score, for which negative values indicate wrong perception and positive values indicate correct perception. Items S26, S9, S11, and S18 exhibit the highest scores (484.5, 445.0, 441.5, and 437.0, respectively), demonstrating that these statements are known to be true by the participants. However, some items did not have such a high score, meaning that people did not know about these topics or had false beliefs about them. The items with the lowest scores were S6 (−10), S8 (25.5), S30 (37.0), and S3 (38.0).

Table A1 in Appendix A presents the correlations between perceptions regarding food myths/facts and the items (variables S1 to S30). Concerning the relation between age and perception of food myths/facts, some weak positive correlations were found to be significant (*p* < 0.01) for items S6 (r_s_ = 0.309), S3 (r_s_ = 0.361) and S1 (r_s_ = 0.286). For variable sex, there were fewer significant correlations (*p* < 0.01), being that the highest value found was for item S23 (r_s_ = 0.208), demonstrating that the correlation is weak. Variables education and environment have very weak influence on the perceptions about food myths and facts. Considering variable marital status, there are some positive significant (*p* < 0.01) correlations with items S6 (r_s_ = 0.266) and S3 (r_s_ = 0.259), even though the associations are weak due to the low values. Being professionally connected to areas, such as nutrition, food, sports, and health also influences the perception cornering food myths and facts. Some significant (*p* < 0.01) weak positive correlations were found between nutrition and items S6 (r_s_ = 0.464), S19 (r_s_ = 0.410), and S20 (r_s_ = 0.396). On the other hand, some significant (*p* < 0.01) weak negative correlations were found between nutrition as items such as S29 (r_s_ = −0.389). As regards to variable food, significant (*p* < 0.01) weak positive correlations were found for items S30 (r_s_ = 0.354), S13 (r_s_ = 0.331), and S6 (r_s_ = 0.326). With respect to sports, a significant (*p* < 0.01) weak positive correlation was found for item S26 (r_s_ = 0.213). Respecting health, some significant (*p* < 0.01) weak positive correlations were found for items S6 (r_s_ = 0.296), S17 (r_s_ = 0.282) and S13 (r_s_ = 0.279). For variable balance diet, there were few significant correlations with perception, and the ones that exist are very weak. Regarding the relation between BMI class and perception of food myths/facts, some significant (*p* < 0.01) weak positive correlations were found such as for item S20 (r_s_ = 0.201).

### 3.4. Level of Knowledge

Concerning the level of knowledge about the 30 questions asked related to food myths and facts, a vast majority of the participants had a high (42.1%) or medium (35.6%) level of knowledge, with some showing a very high level of knowledge (21.7%), a few showing a low level (0.6%), and no respondents showing a very low level.

**Figure 1:**
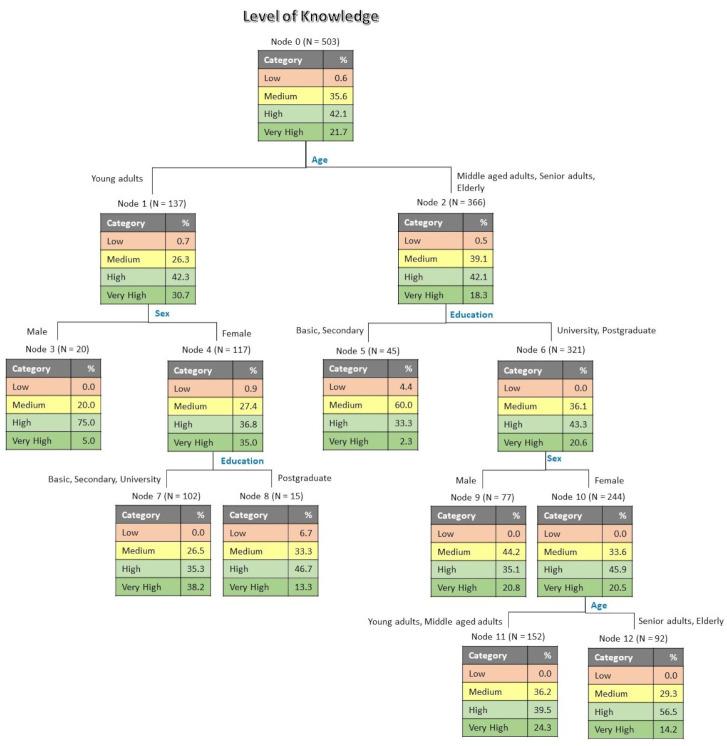
Tree classification for level of knowledge as influenced by sociodemographic variables.

Figure 1 presents the classification tree for level of knowledge as a function of the sociodemographic variables. The obtained tree is four levels deep, with 13 nodes, from which, 7 are terminal. The risk estimate was 0.535 for re-substitution and 0.586 for cross-validation, with standard errors of 0.022 in both cases. According to the results obtained, the first discriminant variable was age, indicating that young adults have higher level of knowledge (30.7% of very high knowledge) in comparison with middle aged adults, senior adults and elderly (18.3% of very high knowledge). For young adults, the next discriminating variable was sex, showing that female participants are better-informed (35.0% of very high knowledge) than male participants (5.0% of very high knowledge). The education level is the discriminating variable following gender, in which respondents with basic, secondary and university education have superior level of knowledge (38.2% of very high knowledge) than postgraduate respondents (13.3% of very high knowledge).

**Figure 2:**
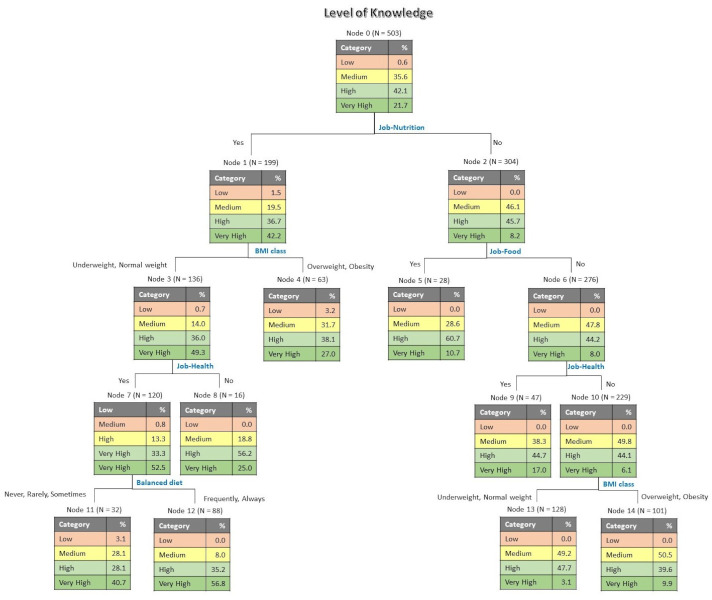
Tree classification for level of knowledge as influenced by other variables, professional areas, BMI, and practice of balanced diet.

The tree in Figure 2 shows the influence of other variables that characterize the sample on the level of knowledge. This tree also has 4 levels and 15 nodes—8 of them terminal. The risk estimate was 0.507 for re-substitution and 0.583 for cross-validation, with standard errors of 0.022 in both cases. The results show that, for the first discriminant variable, participants whose jobs were related to nutrition had very high levels of knowledge (42.2%). The following discriminant was BMI class, in which respondents with overweight or obesity had lower levels of knowledge (27.0% very high) than participants underweight or with normal weight (49.3%). For underweight and normal weight people, the next discriminating variable was professional area of health, showing that those who worked in the health sector had higher levels of knowledge (52.5% very high). A balanced diet was the next discriminant variable, indicating that those participants who always or frequently practiced it had very high levels of knowledge (56.8%) when compared to those who never, rarely, or only sometimes practiced it.

### 3.5. Sources of Information

The main sources of information about nutritional facts were also evaluated. The most frequently used sources were scientific journals (34.4%), DGS website (31.4%), technical books (31.0%), appointments with professionals (medical doctors or nutritionists) (29.2%), and the WHO website (27.2%) (Table 5). Among the least used sources were YouTube channels of influencers (3.0%), natural food stores (7.4%), and pharmacies (8.3%).

The sources with the highest levels of trust were doctor/nutritionist appointments (44.7%), WHO website (42.5%), and DGS website (36.2%). Significant (*p* < 0.01) positive correlations were found between the utilization of information sources and the level of trust, such as, for example, social media (r_s_ = 0.570), YouTube channels of influencers (r_s_ = 0.568), and natural food stores (r_s_ = 0.539) (Table 5).

Some significant (*p* < 0.01) negative correlations were found between knowledge and the utilization of some sources of information, such as natural food stores (r_s_ = −0.355), television (r_s_ = −0.285), and influencers’ YouTube channels (r_s_ = −0.240). Nevertheless, the values are low, which means that the correlations are weak (Table 6). On the other hand, significant (*p* < 0.01) positive correlations were found between knowledge and the use of some sources of information, such as DGS website (r_s_ = 0.320), WHO website (r_s_ = 0.357), and scientific journals (r_s_ = 0.385), although, again, with low values corresponding to weak correlations (Table 6).

Regarding the relation between knowledge and the level of trust in information sources, some negative correlations were found significant (*p* < 0.01) for natural food stores (r_s_ = −0.416), television (r_s_ = −0.266), and influencers’ YouTube channels (r_s_ = −0.234). Again, the correlations are weak because of the low values (Table 6).

## 4. Discussion

Healthy and well-balanced eating habits are important for the maintenance of general health and well-being, prevention of several diseases, and the increase of life expectancy. However, in reality, there are several dietary myths that compromise people’s nutritional knowledge, making it difficult to follow adequate dietary patterns [13]. Hence the relevance of credible and trustworthy sources of nutritional information, in order to optimize the levels of knowledge and, consequently, improve the nutritional behaviors [23]. This study has shown (as previously seen in Table 5) that the main sources used to obtain information about nutrition are scientific journals, DGS website, and technical books. In a study regarding the use of nutritional information, Goodman et al. [10] determined that magazines, newspapers, and books were the third most common source of information, surpassed only by the internet. Even though these are the most utilized sources, doctors and nutritionists are, in this research, the most trusted and reliable. Goodman et al. [10] and Quaidoo et al. [23], in their studies, have also confirmed that healthcare professionals were perceived as the most credible sources of nutritional information.

The grand majority of the participants in this study recognize the importance of water (corresponding to the highest score in Table 4) and adequate hydration, for general health status, since it is involved in the functioning of all cells in the human body. A narrative review has evidenced that dehydration impairs cognitive functions such as attention, motor coordination, and executive functions. Moreover, high fluid intake has been linked to a lower risk of incidence of kidney stones, as well as to maintaining a healthy and functional gastrointestinal system [24,25].

Regarding the statement about drinking milk being bad for health, our results showed that the score was intermediate, revealing that still a lot of participants believe milk to have a negative impact in health. To the matter of milk intake, there is some ambiguity as to which effects dairy products have on health. Milk is an important source of high-quality protein, vitamins and minerals such as Ca, P, Mg, Zn, Se, vitamins A, D, E, and B complex, and bioactive peptides with antimicrobial, antiviral, antifungal, antioxidant, and antibacterial properties [26]. According to a review [27], milk and dairy products have proven to reduce the risk of obesity in children and improve body composition in adults. Additionally, dairy product intake contributes to a reduced risk of type 2 diabetes, bone health maintenance in children and adolescents, and consuming 200 to 300 mL per day does not increase the risk of cardiovascular disease. Milk and dairy probably protect, as well, against colorectal cancer, bladder cancer, gastric cancer, and breast cancer. There is, however, a potential risk for prostate cancer, but the evidence is scarce and inconsistent [27].

One of the items investigated in this survey, which has had a score below the average, is the adoption of a gluten-free diet for better health. This shows that people still have incorrect perceptions about gluten-free diets and think they should be adopted generally. A similar result was reported by Jones [28], according to which 65% of Americans think that gluten-free foods are healthier. In recent years, more people have adopted a gluten-free diet, even though without being diagnosed for celiac disease or non-celiac gluten sensitivity, in which cases, a gluten-free diet is a necessity. There are significant disadvantages in embracing this type of diet, mainly, the lower levels of fiber, iron, zinc, and potassium that products with grains, such as breads and cereals, have, as well as an increased risk of nutritional deficiencies of B vitamins, iron, and other minerals [28].

The statement S30 “All food additives (E’s) are harmful to health” was the third item with the lowest score, corresponding to a generalized wrong perception among the participants in this study. Food additives are substances that are added to products with a specific purpose, such as preservation, coloring, sweetening, and other reasons. All food additives authorized for use are considered safe by the Scientific Committee on Food and/or the European Food Safety Authority. Many of these additives are, in fact, naturally present in foods. As an example tomatoes are rich in lycopene (E160c), oranges have ascorbic acid, or vitamin C (E300), and apples have riboflavin, or vitamin B12 (E101) [29,30].

In general, it was observed that the participants in this study have a wrong perception about the adequacy of eating the fruit before or after a meal (statements S3 and S8). In reality, fruits are an important source of fiber, several vitamins, minerals and phytochemicals, having numerous studies associated the intake of fruits and vegetables with a reduced risk of all-cause mortality, cardiovascular diseases, diabetes, hypertension, and several types of cancer [31,32]. Due to all of these benefits, the World Health Organization recommends the intake of at least 400 g of fruit and vegetables per day [31,32]. In general, the consumption of fresh fruit, prior to or as part of a meal can promote satiety, and reduce hunger and the total energy intake during meals or throughout the day. These properties come, majorly from the high fiber content, which stimulates the release of satiety hormones [32]. Thus, eating fruit right before or after the meal does not bring any benefit apart from those of eating fruit. Only one clinical study has shown that, if fruit is eaten 30 to 120 min prior to the meals, this might have some advantages on maximizing satiety and reduction of energy intake [33].

The item with the lowest score is S6 “Eating carbohydrates at night leads to an increase in weight gain”, demonstrating the incorrect perceptions of the participants about this. Firstly, carbohydrates are not all the same. Some carbohydrates present in vegetables, fruits, and whole-grains are mostly fiber, contrarily to others, such as those in rice, potato, and pasta, which are poorer in dietary fiber. Fiber has demonstrated the ability to facilitate the weight lost, since it helps stimulate satiety hormones, slowing gastric emptying, and reducing appetite, and energy intake [32,34,35]. Secondly, carbohydrates only contribute to weight gain when they are eaten in excessive amounts. When this happens, glucose is transformed into triglycerides, which are then stored in the adipose tissue (fat). Obesity is a disorder in which there is an imbalance between energy intake and expenditure, therefore, the energy present in foods, from all macronutrients, is of extreme importance. Overall, carbohydrates are relevant in the human body, and at night, the most important is to prioritize low density and high fiber foods as well as the amounts consumed. Several studies defend that, for weight loss, the largest meal should be consumed at breakfast or lunch and not in the evening [36,37].

This study has also shown that age is the main discriminant for the level of knowledge exhibited by the participants, with young adults having higher levels of knowledge when compared to other age classes. This might be because in the past years there were attempts to better educate people to eat healthy, as a way to reduce the expenses with health care for patients suffering from non-communicable food-related pathologies. One’s sex is another important discriminant, where women have been shown to have superior levels of knowledge than men. The next discriminant that influences the knowledge is professional area connected with nutrition. Participants with no relation to this field have been shown to have lower levels of knowledge. Furthermore, being underweight and having a normal weight have determined higher levels of knowledge. In work by Guiné et al. [38], also evaluating the sociodemographic factors affecting knowledge about nuts and their health effects, the authors found, as the main discriminants, the level of education, sex, age, exercise, and BMI class. Some of these variables were also found to influence the knowledge about food facts and myths in the present work.

By providing tools to understand what people should eat, this type of work can contribute to the higher literacy of the population if appropriate educational programs and public health strategies are implemented. However, people’s food choices are conditioned by many other factors, some related to personal traits, preferences, or cues, and others associated with social and cultural influences or even economic constraints [39,40,41].

Sociodemographic, anthropometric, and behavioral variables not only influenced the level of knowledge, but also the way participants responded to the questions on myths and facts during this research. The variable age influenced how respondents answered to certain myths/facts such as for example item S1 (Drinking water during meals, contributes to weight gain), item S3 (fruit should be eaten before meals), and item S6 (eating carbohydrates at night leads to an increase in weight gain). As for the professional area, variable nutrition has influenced how participants responded to items such as S6 (eating carbohydrates at night leads to an increase in weight gain), S19 (the alkaline diet allows for balancing the acidity in the blood), and S20 (drinking, while fasting, a glass of water with lemon helps in weight loss). Finally, variable health has influenced items S6 (eating carbohydrates at night leads to an increase in weight gain), S13 (lactose-free foods are better for health and should, therefore, be adopted by all), and S17 (diet should be adapted to a person’s blood group). Age has been reported as a factor affecting people’s care about diet and motivations for food choice. In fact, with increasing age, people tend to think more about health aspects, as a natural way to prevent age-related pathologies or to improve the global health status [17,18].

## 5. Conclusions

This research has shown that, for this sample, the level of knowledge about nutritional facts is quite high. However, this study has also demonstrated that there are still several food myths that need to be debunked through trustworthy information sources, in order to promote healthy, balanced, and appropriate eating behaviors. Aspects that need more attention from public health authorities and educational programs were identified, and will enable the design of more adequate strategies, to improve the level of knowledge of the Portuguese on those aspects in particular. In this way, people could be better educated to make more appropriate food choices and improve their health. This will result, not only in gains, in terms of individual health, but also in public health, and a reduction in the burden associated with non-communicable food-related diseases, such as diabetes, obesity, or heart diseases.

Furthermore, this study concluded that sociodemographic, anthropometric, and behavioral variables not only influenced the level of knowledge, but also the way participants responded to the questions on myths and facts. Hence, despite its limitations, this research provides a scientific perspective on the perception about several food myths and facts. In particular, by highlighting the information that people already have and those aspects that need improvement, the focus of the information campaigns can be directed to target specific myths. Additionally, the sociodemographic characteristics must also be considered when planning information techniques, as the results showed that different sociodemographic groups have different levels of knowledge.

Although providing very useful and new insights into the perceptions about food myths and facts among the Portuguese, this study has some limitations that need to be highlighted. One limitation is related to the nature of the sample, since, as a convenience sample, the results are not statistically representative to be generalized. One other limitation concerns the high levels of education of the participants, which might somehow bias the results. Moreover, the professional areas of a great number of participants are related to food or nutrition; therefore, this could influence their responses.

## Figures and Tables

**Table 1:** Myths and facts used in the questionnaire.

Code	Statement	Nature
S1	Drinking water during meals, contributes to weight gain.	Myth
S2	The digestion process begins in the mouth.	Fact
S3	Fruit should be eaten before meals.	Myth
S4	Egg consumption increases blood cholesterol.	Myth
S5	Drinking milk is bad for health.	Myth
S6	Eating carbohydrates at night leads to an increase in weight gain.	Myth
S7	Fat is important to the human body.	Fact
S8	Fruit should be eaten after meals.	Myth
S9	Fiber intake is important for normal bowel function.	Fact
S10	Gluten-free foods are better for health and should, there-fore, be adopted by all.	Myth
S11	Cheese consumption is bad for memory.	Myth
S12	Coconut oil is healthier than olive oil.	Myth
S13	Lactose-free foods are better for health and should, there-fore, be adopted by all.	Myth
S14	Children have different nutritional needs than those for adults.	Fact
S15	Fruits and vegetables do not contribute to weight gain.	Myth
S16	Normal potatoes are more caloric than sweet potatoes.	Myth
S17	Diet should be adapted to a person’s blood group.	Myth
S18	Not having a balanced and varied diet can lead to the development of multiple diseases.	Fact
S19	The alkaline diet allows balancing the acidity in the blood.	Myth
S20	Drinking, while fasting, a glass of water with lemon helps in weight loss.	Myth
S21	Inadequate eating habits are the third risk factor for the loss of years of healthy life.	Fact
S22	Ingesting high amounts of protein helps in the faster formation of muscles.	Myth
S23	Pregnant women should be eating for two.	Myth
S24	Cold water should not be drunk.	Myth
S25	The day should always start with breakfast.	Fact
S26	Water is essential to the normal function of all organs.	Fact
S27	Soy milk is healthier than cow’s milk.	Myth
S28	Orange should not be eaten at the same time as milk or yogurt.	Myth
S29	Dairy products should be consumed in between two and three portions per day.	Fact
S30	All food additives (E’s) are harmful to health.	Myth

**Table 2:** Sociodemographic characterization of the sample at study.

Variable		N	%
Age group	Young adults (18–30 years)	137	27.2
	Middle aged adults (31–50 years)	216	42.9
	Senior adults (51–65 years)	126	25.0
	Elderly (≥66 years)	24	4.8
Sex	Women	390	77.8
	Men	111	22.2
Education level	Basic	10	2.0
	Secondary	87	17.3
	University	232	46.1
	Post-graduation	174	34.6
Living environment	Urban	287	68.4
	Suburban	57	11.3
	Rural	102	20.3
Marital status	Single	172	34.2
	Married/Union	287	57.1
	Divorced/Separate	38	7.6
	Widowed	6	1.2
Total		503	100

**Table 3:** Anthropometric and behavioral characterization of the sample at study.

**Profession**	**N**	**%**	**Diet**	**N**	**%**
Other	197	39.2	Calorie Restriction	178	35.4
Food	175	34.8	Flexitarianism	43	8.5
Health	168	33.4	Vegetarianism	22	4.4
Nutrition	142	28.2	Fruitarianism	21	4.2
Biology	94	18.7	Crudism	10	2.0
Agriculture	81	16.1	Veganism	8	1.6
Environment	77	15.3	Religious Restriction	7	1.4
Sports	40	8.0			
**BMI percentile**	**BMI (kg/m^2^)**		**BMI Class ^1^**	**%**	
5%	19.1		Underweight	3.4	
10%	20.0		Normal weight	57.5	
25%	21.4		Overweight	32.6	
50%	23.7		Obesity	6.6	
75%	26.3				
90%	28.9				
95%	31.3				

^1^ Underweight (BMI < 18.5 kg/m^2^), normal weight (18.5 ≤ BMI < 25.0 kg/m^2^), overweight (25.0 ≤ BMI < 30.0 kg/m^2^), obesity (BMI ≥ 30 kg/m^2^).

**Table 4:** Item scores regarding the perception for each myth/fact, in descending order.

Items	Mean ± SD ^1^	Sum Score ^2^
S26. Water is essential to the normal function of all organs.	0.96 ± 0.18	484.5
S9. Fiber intake is important for normal bowel function.	0.89 ± 0.25	445.0
S11. Cheese consumption is bad for memory.	0.88 ± 0.31	441.5
S18. Not having a balanced and varied diet can lead to the development of multiple diseases.	0.87 ± 0.30	437.0
S23. Pregnant women should be eating for two.	0.83 ± 0.33	418.0
S14. Children have different nutritional needs than those for adults.	0.82 ± 0.33	410.5
S2. The digestion process begins in the mouth.	0.77 ± 0.49	385.0
S1. Drinking water during meals, contributes to weight gain.	0.70 ± 0.50	351.5
S11. Cheese consumption is bad for memory.	0.88 ± 0.31	441.5
S18. Not having a balanced and varied diet can lead to the development of multiple diseases.	0.87 ± 0.30	437.0
S23. Pregnant women should be eating for two.	0.83 ± 0.33	418.0
S14. Children have different nutritional needs than those for adults.	0.82 ± 0.33	410.5
S2. The digestion process begins in the mouth.	0.77 ± 0.49	385.0
S1. Drinking water during meals, contributes to weight gain.	0.70 ± 0.50	351.5
S25. The day should always start with breakfast.	0.68 ± 0.52	341.5
S21. Inadequate eating habits are the third risk factor for the loss of years of healthy life.	0.66 ± 0.43	330.5
S7. Fat is important to the human body.	0.54 ± 0.53	269.5
S17. Diet should be adapted to a person’s blood group.	0.52 ± 0.58	259.0
S12. Coconut oil is healthier than olive oil.	0.51 ± 0.56	257.0
S13. Lactose-free foods are better for health and should, therefore, be adopted by all.	0.50 ± 0.59	250.5
S5. Drinking milk is bad for health.	0.47 ± 0.59	237.5
S4. Egg consumption increases blood cholesterol.	0.43 ± 0.54	217.5
S10. Gluten-free foods are better for health and should, therefore, be adopted by all.	0.41 ± 0.60	206.5
S27. Soy milk is healthier than cow’s milk.	0.35 ± 0.60	175.0
S20. Drinking, while fasting, a glass of water with lemon helps in weight loss.	0.34 ± 0.61	172.5
S28. Orange should not be eaten at the same time as milk or yogurt.	0.32 ± 0.62	160.5
S19. The alkaline diet allows balancing the acidity in the blood.	0.24 ± 0.59	118.5
S15. Fruits and vegetables do not contribute to weight gain.	0.24 ± 0.59	118.0
S22. Ingesting high amounts of protein helps in the faster formation of muscles.	0.14 ± 0.57	68.5
S29. Dairy products should be consumed in between two and three portions per day.	0.13 ± 0.58	66.5
S24. Cold water should not be drunk.	0.11 ± 0.66	57.0
S16. Normal potatoes are more caloric than sweet potatoes.	0.10 ± 0.68	50.0
S3. Fruit should be eaten before meals.	0.08 ± 0.62	38.0
S30. All food additives (E’s) are harmful to health.	0.07 ± 0.65	37.0
S8. Fruit should be eaten after meals.	0.05 ± 0.58	25.5
S6. Eating carbohydrates at night leads to an increase in weight gain.	−0.02 ± 0.71	−10.0

^1^ Values given as mean and standard deviation, on the scale from −1 (fully incorrect perception) to 1 (fully correct perception). ^2^ The sum score ranges from −503 (fully incorrect perception) to 503 (fully correct perception).

**Table 5:** Expressed opinions and association between the frequency of utilization and level of trust in the information sources.

Information Sources	Frequency of Use (% of Respondents)			Level of Trust (% of Respondents)				Spearman Correlation ^1^
	Never	Few Times	Many Times	No Trust	Some Trust	Much Trust	Full Trust	
YouTube channels of influencers	77.3	19.7	3.0	81.3	18.3	0.4	0	0.568 **
Nutritionist blogs	32.6	46.5	20.9	10.9	60.0	25.6	3.4	0.383 **
DGS website	25.3	43.3	31.4	2.0	22.7	39.2	36.2	0.335 **
Natural food stores	55.9	36.8	7.4	35.0	50.3	11.9	2.8	0.539 **
Scientific journals	27.0	38.6	34.4	2.4	23.7	39.8	34.2	0.362 **
Internet pages	28.2	53.3	18.5	39.0	56.5	4.0	0.6	0.483 **
Social media	54.5	36.6	8.9	67.0	30.8	2.0	0.2	0.570 **
Television	36.2	54.1	9.7	28.8	56.1	13.5	1.6	0.649 **
Technical books	28.6	40.4	31.0	3.0	25.0	38.2	33.8	0.304 **
WHO website	34.2	38.6	27.2	2.4	20.7	34.4	42.5	0.337 **
Pharmacies	58.3	33.4	8.3	13.7	43.7	29.0	3.5	0.341 **
Health centers	38.6	44.9	16.5	4.8	30.2	40.2	24.9	0.301 **
Appointments with Medical doctors/ nutritionists	27.6	43.1	29.2	2.0	18.7	34.6	44.7	0.263 **

^1^ Spearman correlation between frequency of utilization and level of trust. ** Correlation is significant at the 0.01 level.

**Table 6:** Associations between level of knowledge and the frequency of utilization and level of trust in the information sources.

Information Sources	Spearman Correlation between Level of Knowledge and Frequency of Utilization of Information Sources	Spearman Correlation between Level of Knowledge and Level of Trust in the Information Sources
YouTube channels of influencers	−0.240 **	−0.234 **
Nutritionist blogs	−0.063	−0.021
DGS Website	0.320 **	0.357 **
Natural food stores	−0.355 **	−0.416 **
Scientific journals	0.385 **	0.380 **
Internet pages	−0.121 **	0.006
Social media	−0.209 **	−0.161 **
Television	−0.285 **	−0.266 **
Technical books	0.283 **	0.263 **
WHO website	0.357 **	0.342 **
Pharmacies	−0.124 **	−0.179 **
Health centers	0.083	0.086
Appointments with Medical doctors/nutritionists	0.072	0.213 **

** Correlation is significant at the 0.01 level.

## Data Availability

Data are available from the corresponding author upon reasonable request.

## Appendix A

Table A1 presents the Spearman correlations between perception about the food myths/facts and some sociodemographic, anthropometric and behavioral variables.

**Table A1:** Values of Spearman correlation between perception about the food myths/facts and some sociodemographic, anthropometric, and behavioral variables.

Code	Item	Age	Sex	Education	Environment	Marital	Nutrition	Food	Sport	Health	Bal. Diet	BMI Class
S1	Drink water at meals	0.286 **	0.037	0.049	−0.039	0.172 **	0.286 **	0.281 **	0.045	0.183 **	−0.106 *	0.113 *
S2	Beginning of digestion	0.033	−0.002	0.089 *	0.032	−0.028	−0.202 **	−0.158 **	−0.024	−0.167 **	0.130 **	0.001
S3	Fruit before meals	0.361 **	−0.013	0.083	0.038	0.259 **	0.307 **	0.186 **	0.046	0.113 *	−0.042	0.134 **
S4	Eggs and cholesterol	0.041	0.089 *	−0.035	0.143 **	0.042	0.095	0.064	−0.084	0.018	−0.128 **	−0.060
S5	Milk is bad for health	0.143 **	0.044	−0.083	0.012	0.104 *	0.282 **	0.326 **	−0.026	0.214 **	−0.046	0.101 *
S6	Carbohydrates at night and weight	0.309 **	−0.002	0.061	0.011	0.266 **	0.464 **	0.299 **	0.087	0.296 **	−0.144 **	0.158 **
S7	Importance of fat	−0.195 **	−0.042	0.049	0.043	−0.174 **	−0.369 **	−0.331 **	−0.075	−0.225 **	0.132 **	−0.117 **
S8	Fruit after meals	−0.138 **	0.130 **	0.008	0.047	−0.141 **	−0.008	−0.042	−0.011	0.053	−0.012	−0.024
S9	Fiber and bowel function.	0.164 **	−0.069	0.108 *	−0.006	0.103 *	−0.226 **	−0.188 **	−0.043	−0.114 *	0.136 **	0.008
S10	Gluten-free foods are better for all	0.148 **	0.099 *	−0.073	0.002	0.107 *	0.275 **	0.203 **	−0.052	0.177 **	−0.086	0.133 **
S11	Cheese is bad for memory	−0.071	0.011	−0.086	0.007	−0.049	0.040	0.015	−0.028	0.112 *	0.006	−0.001
S12	Coconut oil versus olive oil	−0.066	0.072	−0.023	−0.091 *	−0.001	0.306 **	0.261 **	0.033	0.182 **	−0.117 **	0.084
S13	Lactose-free foods are better for all	0.120 **	0.059	−0.144 **	−0.029	0.070	0.363 **	0.331 **	−0.007	0.279 **	−0.056	0.098 *
S14	Children’s nutritional needs	−0.066	−0.109 *	−0.007	0.001	−0.045	−0.147 **	−0.118 *	−0.004	−0.103 *	0.026	0.004
S15	Fruits/vegetables and weight gain	0.061	0.052	−0.090 *	−0.017	0.014	0.133 **	0.058	0.041	0.085	−0.071	0.107 *
S16	Sweet potatoes have less calories	0.180 **	0.042	0.060	0.008	0.149 **	0.376 **	0.295 **	0.119 *	0.220 **	−0.128 **	0.039
S17	Diet and blood group	0.070	0.072	0.023	−0.030	0.049	0.335 **	0.234 **	0.038	0.282 **	−0.101*	0.110 *
S18	Balanced varied diet and diseases	0.047	−0.130 **	0.102 *	0.028	0.061	−0.133 **	−0.099 *	0.107 *	−0.026	0.096 *	−0.009
S19	Alkaline diet and blood acidity	0.211 **	0.129 **	−0.043	−0.014	0.175 **	0.410 **	0.321 **	0.028	0.258 **	−0.058	0.145 **
S20	Water with lemon and weight loss	0.238 **	0.051	−0.030	0.019	0.218 **	0.396 **	0.313 **	0.030	0.247 **	−0.149 **	0.201 **
S21	Eating habits and risk of disease	0.160 **	−0.064	0.026	−0.037	0.132 **	−0.079	−0.056	−0.096	−0.068	0.077	0.046
S22	Protein and muscle formation	−0.087	0.049	−0.023	−0.032	−0.099 *	0.128 *	0.076	−0.017	0.174 **	−0.016	0.044
S23	Pregnant women eating for two	−0.106 *	0.208 **	−0.036	0.070	−0.194 **	0.191 **	0.086	0.037	0.133 *	−0.073	0.029
S24	Cold water should not be drunk	0.153 **	0.022	−0.086	−0.035	0.058	0.271 **	0.184 **	−0.073	0.208 **	−0.016	0.022
S25	Importance of breakfast	0.191 **	0.087	−0.043	−0.021	0.158 **	0.223 **	0.163 **	0.083	0.090	−0.087	0.092 *
S26	Water is essential for organs	0.115 **	−0.065	0.051	0.007	0.046	0.125 *	0.062	0.213 **	0.090	0.082	−0.053
S27	Soy milk is healthier	0.051	0.058	−0.139 **	−0.012	0.045	0.359 **	0.313 **	−0.005	0.228 **	−0.135 **	0.117 **
S28	Orange and dairy products	0.040	−0.006	−0.034	−0.076	0.016	0.227 **	0.210 **	0.007	0.186 **	−0.010	0.067
S29	Portions of dairy products	−0.117 **	−0.152 **	−0.004	−0.020	−0.094 *	−0.389 **	−0.321 **	−0.104	−0.273 **	0.162 **	−0.057
S30	Food additives (E’s) and health	0.135 **	−0.059	−0.043	−0.121 **	0.118 **	0.356 **	0.354 **	0.060	0.141 **	−0.090 *	0.057

* Correlation significant at 0.05 level. ** Correlation significant at 0.01 level.
